# Postmortem Temporal Changes in Liver and Spleen Stiffness: Evaluation with Shear Wave Elastography in a Rat Model

**DOI:** 10.3390/diagnostics15080958

**Published:** 2025-04-10

**Authors:** Ismail Taskent, Selçuk Başer, Bunyamin Ece, Serbülent Kılıç, Ugur Akpulat, Irfan Cinar, Nurtaç Sarıkaş

**Affiliations:** 1 Department of Radiology, Kastamonu University, 37150 Kastamonu, Turkey; bunyaminece@hotmail.com; 2 Department of Radiology, Kastamonu Training and Research Hospital, 37150 Kastamonu, Turkey; slck71bsr@gmail.com; 3 Department of Forensic Medicine, Kastamonu University, 37150 Kastamonu, Turkey; kilicserbulentmd@gmail.com; 4 Department of Medical Biology, Kastamonu University, 37150 Kastamonu, Turkey; uakpulat@kastamonu.edu.tr; 5 Department of Pharmacology, Kastamonu University, 37150 Kastamonu, Turkey; atairfan.nar@gmail.com; 6 Department of Pathology, Kastamonu University, 37150 Kastamonu, Turkey; nsarikas@kastamonu.edu.tr

**Keywords:** liver stiffness, spleen stiffness, shear wave elastography, rat model, postmortem

## Abstract

**Background/Objectives**: Postmortem changes in tissue stiffness and organ morphology are critical for forensic medicine and pathology. Shear wave elastography (SWE) has emerged as a non-invasive tool to assess tissue stiffness, yet its potential for postmortem interval estimation remains underexplored. While previous studies have demonstrated early postmortem alterations in tissue elasticity, the temporal progression of these changes in different organs is not fully understood. This study aims to investigate the temporal changes in liver and spleen stiffness during the postmortem period using SWE and to evaluate the predictive potential of elastographic parameters for postmortem interval estimation. **Methods**: Twelve male Sprague–Dawley rats were sacrificed via cervical dislocation following deep anesthesia. Postmortem liver and spleen measurements, including longitudinal and short diameters and SWE values (kPa), were recorded at 0, 2, 4, 6, 9, 12, 18, 24, and 36 h. All elastographic measurements were obtained using a 5 mm circular region of interest (ROI) for the liver and a 3 mm ROI for the spleen. Changes over time were analyzed using repeated measures ANOVA, with post hoc Bonferroni corrections applied where necessary. Additionally, Receiver Operating Characteristic (ROC) curve analysis and binary logistic regression analysis were performed to assess the predictive accuracy of SWE parameters in estimating postmortem time. **Results**: Postmortem liver and spleen stiffness exhibited a significant declining trend over time (*p* < 0.001, η^2^ = 0.749 and η^2^ = 0.810, respectively). Liver and spleen dimensions initially increased, reaching peak values around 6 h, followed by a gradual reduction. ROC analysis demonstrated that spleen SWE (AUC = 0.917) and liver SWE (AUC = 0.845) were the strongest predictors of early postmortem time. Binary logistic regression further confirmed that liver and spleen SWE were statistically significant predictors of postmortem time (*p* = 0.006 and *p* = 0.020, respectively). **Conclusions**: This study provides evidence that postmortem liver and spleen stiffness decline progressively over time, while organ dimensions exhibit a biphasic pattern. Elastographic parameters, particularly SWE values, demonstrated strong predictive accuracy in estimating early postmortem intervals. These findings suggest that SWE may serve as a valuable imaging modality for forensic applications, providing objective insights into postmortem biomechanical changes and time-of-death estimation. Further research should explore the applicability of SWE in different tissue types and under varying environmental conditions.

## 1. Introduction

Changes in the biophysical properties of organs during the postmortem process are a significant research topic in the fields of forensic medicine, pathology, and biomedical imaging [1,2]. In particular, alterations in the stiffness of organs such as the liver and spleen can contribute to understanding tissue degradation in the postmortem period [3,4,5].

Following death, organs undergo a range of biochemical and structural changes primarily driven by metabolic exhaustion. Rapid depletion of ATP disrupts ionic gradients, impairs membrane transport, and leads to cytoplasmic swelling, mitochondrial dysfunction, and eventual autolysis. These alterations affect the mechanical properties of soft tissues such as the liver and spleen, which may result in measurable changes in tissue stiffness [6,7]. Advanced imaging techniques such as magnetic resonance elastography (MRE), diffusion-weighted imaging (DWI), and shear wave elastography (SWE) enable the objective evaluation of these changes [8,9]. SWE, as a quantitative and non-invasive imaging technique, has the potential to detect these subtle postmortem changes. Understanding how tissue stiffness evolves in response to biochemical degradation could enhance the accuracy of postmortem interval (PMI) estimation in forensic settings. However, studies in this field are still limited, and there is insufficient data, particularly regarding the temporal dynamics of postmortem elastography measurement.

Previous studies indicate that significant biophysical changes occur in the liver and spleen during the early hours of the postmortem process. Garczyńska et al. detected significant changes in liver stiffness and water diffusion during the postmortem process, reporting that these changes were particularly pronounced within the first 2 h [8]. Similarly, Keller et al. identified significant changes in liver tissue during the early postmortem period using MR diffusion measurements [9]. Donaldson and Lamont, on the other hand, emphasized that postmortem biochemical markers could be used to estimate organ functions [6].

Ultrasound elastography is a non-invasive imaging technique that provides information about tissue stiffness, and it has gained increasing attention in recent years for its clinical and experimental applications. Two main types of elastography are currently used: strain elastography and shear wave elastography (SWE). Strain elastography is a qualitative or semi-quantitative method that relies on manual compression to evaluate tissue deformation, and it is often more suitable for focal lesions. In contrast, SWE is a quantitative technique based on the measurement of shear wave velocity, offering objective and reproducible stiffness values. It has shown particular promise in assessing diffuse changes in soft tissues and in studies requiring longitudinal measurements [10].

SWE is increasingly being used in both clinical and experimental studies to assess liver and spleen stiffness. The majority of existing studies have focused on the role of elastography in the diagnosis of liver fibrosis and portal hypertension [11,12]. However, research examining the elastography changes in the liver and spleen during the postmortem process is quite limited. Chen et al. demonstrated a significant decrease in spleen stiffness postmortem and suggested that these changes are largely related to portal hypertension but not directly caused by postmortem alterations [13].

Elastography is an important tool for understanding the changes occurring in tissue biomechanics during the postmortem process [6,14]. Bertalan et al. revealed, using MRE, that biomechanical changes in hypoxic and dying brain tissue follow a predictable pattern [15]. Additionally, Sugimoto et al. analyzed viscosity changes in rat livers and determined that inflammation increases tissue viscosity [16]. These studies are significant in evaluating the reliability of elastography measurements during the postmortem process. However, there is no study that systematically assesses how liver and spleen stiffness change over time in the postmortem process using SWE.

The aim of this study is to evaluate changes in organ size and elasticity, as measured by SWE, in rat liver and spleen tissues during the postmortem process, and to determine the potential of these parameters in estimating the time of death.

## 2. Materials and Methods

### 2.1. Ethical Approval

Our study was designed to evaluate postmortem changes in liver and spleen elasticity and organ dimensions using SWE. The study was approved by the local animal experiments ethics committee on 11 March 2024 (Approval No: 06). Additionally, confirmation was obtained from this committee stating that no additional permission was required for this study (Approval No: 32/12 November 2024). The experiment was conducted under controlled room conditions (23 ± 1 °C and 35–40% humidity), and serial abdominal ultrasound and SWE measurements were performed at predefined time intervals. A total of 12 male Sprague–Dawley rats (8 weeks old, 250 ± 20 g) were included in the study, and measurements were obtained following humane euthanasia.

### 2.2. Sacrifice Procedure

Rats were sedated via intraperitoneal injection of ketamine (90 mg/kg) and xylazine (5–10 mg/kg) to ensure adequate anesthesia and minimize pain and discomfort during procedures. The depth of anesthesia was assessed by the absence of reflex responses, and all procedures were performed in accordance with ethical guidelines to ensure animal welfare. Following sedation, the rats were humanely euthanized using the cervical dislocation method, which is a rapid and ethically approved technique.

### 2.3. Ultrasound Evaluation

To evaluate elasticity and dimensional changes in liver and spleen tissues during the postmortem period, measurements were obtained from all rats at 0, 2, 4, 6, 9, 12, 18, 24, and 36 h postmortem.

Organ measurements and SWE assessments were performed using a high-frequency linear probe (L15–3 MHz) attached to a Resona R9 ultrasound system (Mindray Medical, Shenzhen, China). All measurements were conducted with the rats placed in the supine position. The most suitable acoustic window was identified for each organ to ensure artifact-free imaging. For liver and spleen evaluations, the transducer was positioned perpendicular to the surface, and imaging was primarily performed in the transverse plane to obtain consistent parenchymal sections.

**Figure 1:**
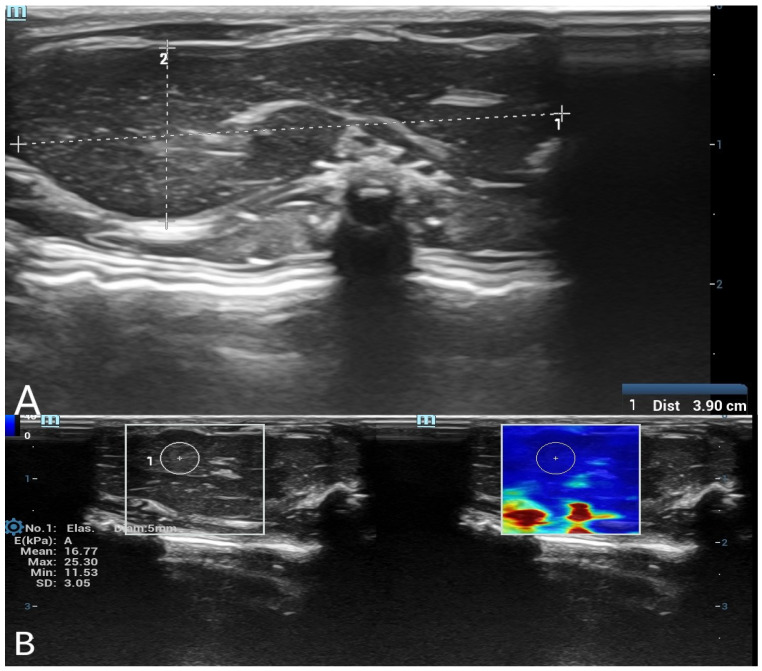
(**A**) B-mode ultrasound measurement of liver long and short diameters. A grayscale ultrasound image showing the measurement of liver long and short diameters. The calipers indicate the dimensions of the liver in the postmortem assessment. (**B**) Shear wave elastography (SWE) measurement of liver stiffness with a 5 mm region of interest (ROI). The left panel displays the grayscale ultrasound image of the liver, while the right panel shows the corresponding SWE color map. A 5 mm circular ROI is placed in the liver parenchyma to obtain quantitative stiffness values in kilopascals (kPa).

**Figure 2:**
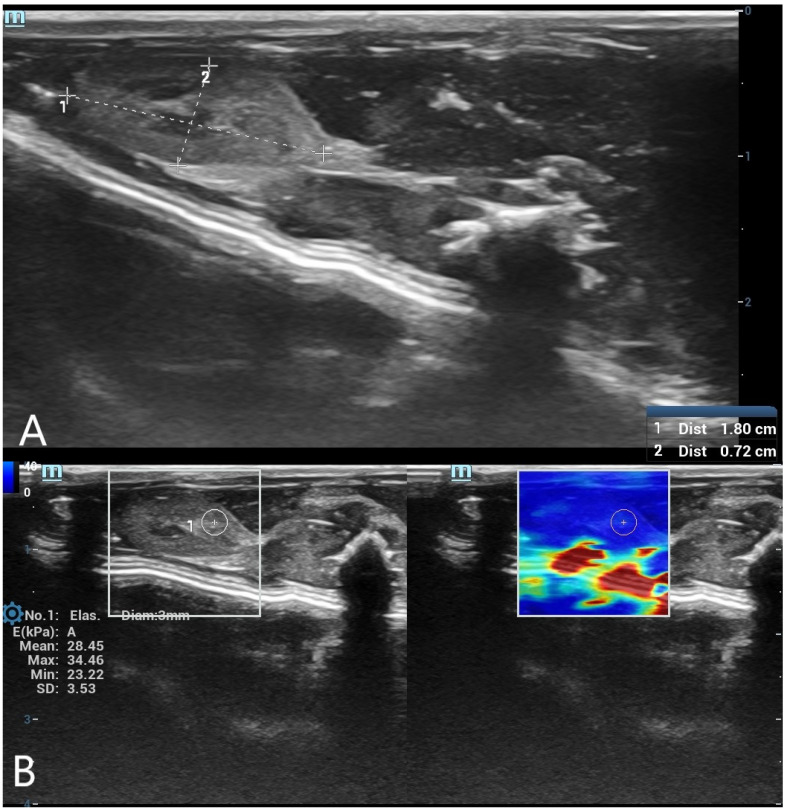
(**A**) B-mode ultrasound measurement of spleen long and short diameters. A grayscale ultrasound image illustrating the measurement of spleen long and short diameters using electronic calipers. These dimensions were used to evaluate postmortem organ changes. (**B**) Shear wave elastography (SWE) measurement of spleen stiffness with a 3 mm region of interest (ROI). The left panel presents the grayscale ultrasound image of the spleen, while the right panel contains the SWE color-coded stiffness map. A 3 mm circular ROI is positioned within the spleen tissue to assess postmortem stiffness changes.

SWE measurements were acquired by a radiologist with over 8 years of experience in ultrasonography. A circular region of interest (ROI) with a diameter of 5 mm was placed centrally within the liver parenchyma, and a 3 mm ROI was used for the spleen parenchyma. ROIs were placed carefully in homogeneous tissue areas, avoiding visible ducts, vessels, or peripheral artifacts. The measurement protocol was standardized based on consensus among three radiologists before the study began. At each time point, three separate measurements were obtained and averaged. Only SWE maps without significant artifacts were included in the analysis. SWE results were recorded in kilopascals (kPa), and minimum, maximum, mean, and standard deviation (SD) values were calculated for each organ (Figure 1 and Figure 2).

### 2.4. Statistical Analysis

Statistical analyses were performed using SPSS v.23 (SPSS, Chicago, IL, USA). Continuous variables were reported as mean ± standard deviation (SD) or median (minimum-maximum). To evaluate the changes in liver and spleen long diameter, short diameter, and elasticity values over time, repeated measures ANOVA was performed. To assess the overall effect of time on dependent variables, Pillai’s Trace, Wilks’ Lambda, Hotelling’s Trace, and Roy’s Largest Root tests were applied. For statistically significant findings, Bonferroni-adjusted post hoc tests were used for pairwise comparisons between time points. Trend analysis was conducted to examine linear and quadratic trends over time. Additionally, ROC analysis and binary logistic regression analysis were conducted to predict the postmortem period. For ROC analysis, the postmortem interval was categorized into four time groups: 0–4, 6–9, 12–18, and 24–36 h. The 0–4 h group, representing the early postmortem window with the most pronounced biological changes, was designated as the positive state to assess the diagnostic performance of elastographic measurements. Mean values within each group were used for analysis to reduce intra-group variability and support statistical consistency. A *p*-value < 0.05 was considered statistically significant in all tests.

## 3. Results

In this study, postmortem changes in liver and spleen measurements in rats were comprehensively evaluated, and the effect of time on organ size and elasticity values was statistically analyzed. Repeated measures ANOVA was applied to assess changes over time, and multiple comparisons were performed to identify significant differences between specific time points. The findings indicate that significant changes occurred in organ size and elasticity values during the postmortem period.

In our study, baseline (0 h) measurements were evaluated separately for all rats. The mean liver long diameter (LLD) was 38.00 ± 3.85 mm, and the liver short diameter (LSD) was 15.67 ± 1.51 mm. The mean spleen long diameter (SLD) was 17.08 ± 1.61 mm, while the spleen short diameter (SSD) was recorded as 7.67 ± 0.62 mm. When assessed using shear wave elastography (SWE), the mean liver elasticity (LE) was found to be 32.35 ± 5.88 kPa, and the spleen elasticity (SE) was measured as 33.09 ± 4.83 kPa. Measurements taken over time revealed that both dimensional changes and elasticity values followed a specific trend throughout the postmortem period (Table 1).

**Figure 3:**
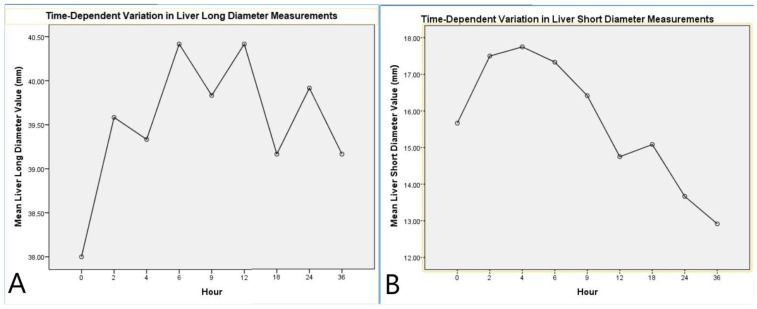
Time-dependent variation in liver diameter measurements. (**A**) Mean liver long diameter values measured at different postmortem time points. (**B**) Mean liver short diameter values measured at different postmortem time points. Both graphs illustrate the dynamic changes in liver dimensions over time, showing initial fluctuations followed by a trend toward stabilization or reduction.

Repeated measures ANOVA analysis for the LLD indicated that the time factor did not have a significant overall effect on the LLD (F(8,88) = 1.337, *p* = 0.236, η^2^ = 0.108). However, pairwise comparisons between time points revealed a statistically significant increase between time 1 and time 4 (*p* = 0.045). The LLD reached its peak at the 6th hour, followed by a gradual decrease (Table 2, Figure 3).

Analysis of the LSD indicated a significant effect of the time factor (F(8,88) = 22.616, *p* < 0.001, η^2^ = 0.673). Multivariate tests (Pillai’s Trace, Wilks’ Lambda, Hotelling’s Trace, and Roy’s Largest Root) also confirmed the significant impact of time on the LSD (*p* = 0.011, η^2^ = 0.965). Pairwise comparisons showed a marked increase in the short diameter during the early postmortem period, followed by stabilization. Linear (*p* < 0.001, η^2^ = 0.860) and quadratic trend analyses (*p* < 0.001, η^2^ = 0.702) supported the temporal dynamics of this change (Table 3, Figure 3).

**Figure 4:**
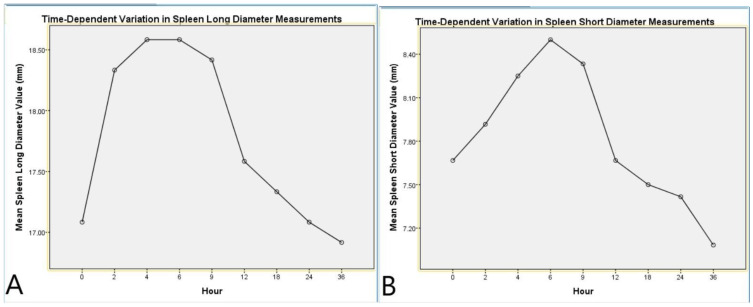
Time-dependent variation in spleen diameter measurements. (**A**) Mean spleen long diameter values measured at different postmortem time points. (**B**) Mean spleen short diameter values measured at different postmortem time points. Both graphs demonstrate the dynamic postmortem changes in spleen dimensions, with an initial increase followed by a gradual decline over time.

Analysis of the SLD revealed a significant effect of the time factor (F(8,88) = 8.469, *p* < 0.001, η^2^ = 0.435). Post hoc pairwise comparisons showed significant differences between baseline (0 h) and the measurements at 2, 4, 6, and 9 h postmortem (*p* < 0.05). Quadratic trend analysis indicated that the SLD initially increased slightly but gradually exhibited a decreasing trend over time (*p* < 0.001, η^2^ = 0.751, Table 4, Figure 4).

Analysis of the SSD also revealed a significant effect of the time factor (F(8,88) = 5.984, *p* < 0.001, η^2^ = 0.352). Post hoc pairwise comparisons demonstrated significant differences between baseline (0 h) and the measurements at 4, 6, and 9 h postmortem (*p* < 0.05). Quadratic trend analysis demonstrated a distinct change in the SSD throughout the postmortem period (*p* < 0.001, η^2^ = 0.707, Table 5, Figure 4).

**Figure 5:**
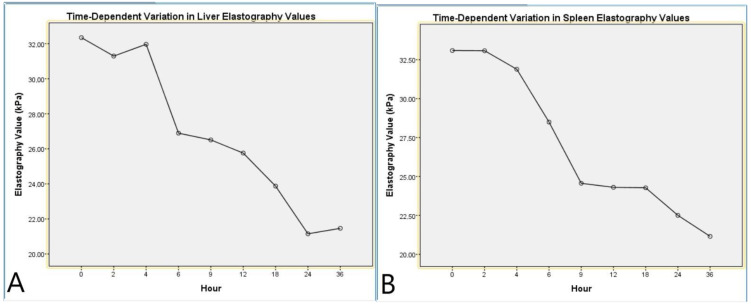
Time-dependent variation in liver and spleen elastography values. (**A**) Mean liver elastography values measured at different postmortem time points using shear wave elastography. (**B**) Mean spleen elastography values measured at different postmortem time points using shear wave elastography. Both graphs illustrate a progressive decrease in tissue stiffness over time, reflecting postmortem tissue softening.

Repeated measures ANOVA analysis for LE indicated a significant effect of the time factor on liver stiffness (F(8,88) = 12.7, *p* < 0.001, η^2^ = 0.536). Pairwise comparisons revealed significant differences between hour 1 and hour 4 (*p* = 0.003) and between hour 1 and hour 8 (*p* < 0.001). Linear trend analysis demonstrated a notable decrease in elasticity values as the postmortem process progressed (*p* < 0.001, η^2^ = 0.749, Table 6, Figure 5).

The repeated measures ANOVA analysis for SE demonstrated a significant effect of the time factor on spleen stiffness (F(8,88) = 12.37, *p* < 0.001, η^2^ = 0.529). Notably, significant differences were observed between hour 1 and hour 5 (*p* = 0.001) and between hour 1 and hour 9 (*p* < 0.001). Linear trend analysis revealed a gradual decline in spleen elasticity over time, indicating a specific pattern throughout the postmortem process (*p* < 0.001, η^2^ = 0.810). These findings suggest that the biomechanical properties of the spleen undergo distinct alterations during the postmortem period (Table 7, Figure 5).

To estimate postmortem time, the predictive value of liver and spleen morphological and elastographic parameters was assessed using Receiver Operating Characteristic (ROC) curve analysis. The time variable was categorized into four groups: 0–4 h (Time 1), 6–9 h (Time 2), 12–18 h (Time 3), and 24–36 h (Time 4). ROC analysis was conducted using Time 1 as the reference category (positive state) to evaluate the ability of each parameter to distinguish early postmortem times. The results demonstrated that spleen elastography (SEV, AUC = 0.917) and liver elastography (LEV, AUC = 0.845) exhibited the highest discriminatory power in differentiating early postmortem times from later stages. Among the morphological parameters, the spleen short diameter (LSD, AUC = 0.794) showed moderate predictive value, while the liver long diameter (LLD, AUC = 0.425) had limited predictive capacity.

**Figure 6:**
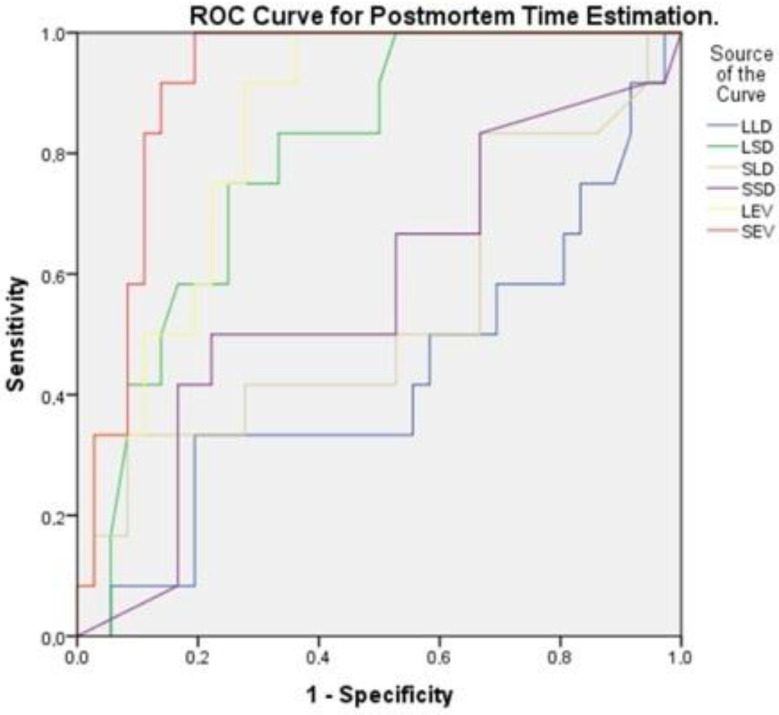
Receiver Operating Characteristic (ROC) curve for postmortem time estimation. ROC curve analysis for differentiating early postmortem time based on liver and spleen morphological and elastographic parameters. The area under the curve values indicate that spleen elastography (red) and liver elastography (purple) are the highest.

Additionally, in the binary logistic regression analysis, the dependent variable was defined based on the time classification used in the ROC analysis. Specifically, the 0–4 h interval (Time 1) was coded as “1” (positive class), while the 6–9, 12–18, and 24–36 h intervals were coded as “0” (negative class). This classification was used to assess the predictive performance of elastography parameters in distinguishing early postmortem cases. The model was found to be statistically significant (χ^2^ = 24.811; *p* < 0.001) and showed good explanatory power (Nagelkerke R^2^ = 0.538). The overall classification accuracy was 75%, with a sensitivity of 79.2% and a specificity of 70.8%. These findings indicate that both spleen and liver SWE values serve as strong predictors for early postmortem interval estimation (*p* = 0.006 and *p* = 0.020, respectively) (Table 8, Figure 6).

## 4. Discussion

In this study, the temporal changes in liver and spleen elasticity values and organ sizes during the postmortem process in rats were systematically evaluated. Our findings demonstrated that, as the postmortem process progressed, both liver and spleen elasticity values decreased, whereas organ sizes initially exhibited a significant increase, followed by a reduction in later stages. Significant changes were detected in liver short diameter and spleen dimensions, with linear and quadratic trend analyses confirming that these changes followed a systematic pattern. Notably, organ sizes peaked around the 6th hour and subsequently showed a decreasing trend. Furthermore, the results of the ROC analysis and logistic regression analysis indicated that spleen and liver elasticity values exhibited high accuracy in determining the early postmortem period, suggesting that elasticity measurements may serve as a more reliable indicator for postmortem time estimation compared to organ size measurements.

Previous studies have demonstrated that significant biophysical changes occur in the liver during the early postmortem period. Studies on bovine liver parenchyma also reported significant reductions in failure strain with increasing postmortem time, particularly when stored in saline, highlighting the progressive weakening of liver tissue [17]. Additionally, a recent canine study demonstrated that biochemical markers such as LDH and AST in the liver exhibited significant alterations within the first 4–6 h postmortem, accompanied by clear histopathological changes. These findings further support the sensitivity of hepatic tissue to early postmortem degradation and are consistent with the marked reduction in elasticity observed in our rat model [18]. In a study by Garczyńska et al., changes in the biophysical properties of liver tissue during the postmortem process were assessed using magnetic resonance elastography (MRE) and diffusion-weighted imaging (DWI). Their findings indicated significant alterations in the early postmortem period (first 2 h), followed by a relatively stable phase until the 10th hour, after which tissue degradation progressively increased [8]. These changes have been associated with perfusion loss in the tissue and increased cellular degeneration as the postmortem process progresses. Similarly, our study observed a significant decrease in elasticity values over time, becoming more pronounced after the 6th hour. Consistent with the findings of Garczyńska et al. [8], our study also demonstrated that the mechanical properties of tissues deteriorate during the postmortem period.

Postmortem biochemical changes can have a direct impact on tissue integrity and mechanical properties. The continuation of cellular degradation and metabolic processes after death can influence tissue fluid dynamics, leading to a gradual decrease in elasticity values over time. In particular, the loss of cell membrane integrity and interstitial fluid shifts may contribute to the reduction in tissue stiffness. Additionally, the progression of cellular autolysis and enzymatic processes can result in predictable alterations in tissue biomechanics during the postmortem period [6,16]. Bertalan et al. investigated postmortem elasticity changes in the brain and observed a significant decrease in the early hours, which they attributed to cellular degradation [15]. Similarly, in our study, liver and spleen elasticity values decreased over time, which may be associated with the degradation of the extracellular matrix and the disruption of tissue integrity during the postmortem process.

In a study by Sayers et al., endotoxin was injected into rats 40 min before death, and endotoxin levels in the organs were assessed during the postmortem process [14]. In this study, endotoxin levels were reported to show a significant increase at the 6th hour, remaining relatively stable after the 30th hour. Similarly, in our study, liver and spleen elastography values demonstrated a significant decrease at the 6th hour and remained low at the 36th hour. This reduction in tissue stiffness may reflect changes in the mechanical properties of tissues during the postmortem process. Additionally, significant expansion in liver and spleen long and short diameters was observed at the 6th hour, but this increase was followed by a decreasing trend in later hours. These findings suggest that the elevated endotoxin levels in the early postmortem period may be associated with a transient expansion in tissue volume. However, different methodologies and the effects of the postmortem process on tissue morphology should be considered. The obtained results emphasize the importance of integrating both biochemical and imaging methods in evaluating postmortem changes.

Changes in organ size during the postmortem process can also be explained at the histological level. In a study by Tomita et al., mitochondrial alterations and cellular swelling were reported to occur in the early hours of the postmortem process at the ultrastructural level [7]. Keller et al. reported that the liver exhibited increased water mobility during the postmortem process as assessed by MR diffusion-weighted imaging, which subsequently stabilized after a certain period [9]. Similarly, in our study, a significant increase in liver and spleen size was observed in the initial hours, reaching a maximum level around the 6th hour, followed by a gradual decrease. This suggests that transient organ enlargement occurs due to cellular swelling, fluid shifts, and tissue edema in the early postmortem period, while subsequent cellular degeneration and fluid loss contribute to organ shrinkage over time. The mitochondrial changes and cellular degeneration described in a study by Tomita et al., along with the increase in water mobility observed by Keller et al. during the postmortem process, align with our findings on organ size fluctuations. These results suggest that changes in organ volume during the postmortem period may be linked to histopathological mechanisms at the cellular level.

In a study by Leong et al. (2023), the temporal changes in tissue stiffness during the postmortem period were evaluated using the SWE method in canines, with elasticity measurements performed on the liver, spleen, kidneys, and muscle tissue. A transient increase in elasticity values was observed in the early postmortem period, followed by a marked decreasing trend starting from the 6th hour. The study emphasized that postmortem elasticity measurements could contribute to estimating the time of death [19]. Similarly, in our study, it was determined that liver and spleen elasticity values in rats significantly decreased as the postmortem process progressed. However, while Leong et al.’s study observed a transient increase in elasticity values during the early postmortem period, our study demonstrated a more stable trend in elasticity values from the early phase, followed by a significant decline after the 6th hour. Our findings further support the effectiveness of SWE in detecting tissue stiffness changes during the postmortem process and highlight its potential as a valuable tool for estimating the time of death.

The findings of this study suggest that SWE may serve as a useful complementary tool in forensic practice for estimating the postmortem interval. As a non-invasive, quantitative imaging method, SWE enables the detection of early tissue stiffness changes, particularly within the first 4 h after death. This early sensitivity could assist forensic experts when conventional methods are inconclusive. However, SWE measurements can be affected by factors such as body temperature, cause of death, and environmental conditions. Therefore, careful interpretation is essential. Future studies involving human cadavers and standardized protocols are needed to validate these results and explore the broader forensic applicability of SWE.

Our study has some limitations. The measurements were performed by a single radiologist, which restricts the evaluation of inter-observer variability. Additionally, factors such as tissue perfusion or biochemical processes that may contribute to changes in elasticity were not directly examined. Further studies using different methodologies and multicenter research are needed to comprehensively evaluate elasticity changes during the postmortem process.

## 5. Conclusions

This study revealed a significant decrease in liver and spleen elasticity values during the postmortem process, while organ dimensions initially exhibited a temporary increase followed by a reduction. ROC analysis demonstrated that spleen and liver elasticity values showed high accuracy in predicting the early postmortem period. Logistic regression analysis confirmed that elasticity variables were significant predictors of postmortem time estimation. These findings suggest that biomechanical changes in organs during the postmortem process could serve as important indicators for estimating the time of death. Compared to organ size measurements, elasticity assessments may provide a more reliable marker for postmortem time estimation. Future studies should further investigate the relationship between SWE measurements and postmortem biophysical changes across different tissue types.

## Figures and Tables

**Table 1:** Baseline liver and spleen measurements in rats at 0 h.

Rat No	LLD (mm)	LSD (mm)	SLD (mm)	SSD (mm)	Liver SWE (kPa)	Spleen SWE (kPa)
1	41.1	15.2	20.4	8.1	22.76	38.61
2	38.7	14.1	15.2	8.3	25.92	34.41
3	40.3	17.4	17.3	7.4	30.60	34.22
4	42.2	17.2	17.5	8.2	34.22	29.61
5	44.4	18.5	19.1	7.4	32.35	28.26
6	36.3	14.4	17.3	7.3	39.95	39.58
7	33.7	15.6	18.7	7.7	30.86	41.38
8	34.6	13.5	14.9	6.6	39.42	31.58
9	29.8	15.8	15.5	7.8	41.56	28.53
10	39.7	15.6	15.7	6.6	31.62	34.24
11	37.3	16.9	16.8	8.7	25.73	30.24
12	37.9	13.8	16.6	7.9	33.20	26.43
Mean ± SD	38.0 ± 3.85	15.67 ± 1.51	17.08 ± 1.61	7.67 ± 0.62	32.35 ± 5.88	33.09 ± 4.83

LLD: liver long diameter; LSD: liver short diameter; SLD: spleen long diameter; SSD: spleen short diameter; SWE: shear wave elastography; kPa: kilopascal; mm: millimeter.

**Table 2:** Liver long diameter measurements at different postmortem time points.

Hour	Mean LLD (mm) ± SD	95% CI	*p* Value
0	38.00 ± 3.85	35.56–40.44	Reference
2	39.58 ± 3.82	37.15–42.01	0.095
4	39.33 ± 4.68	36.36–42.31	0.333
6	40.42 ± 3.75	38.03–42.80	**0.045**
9	39.83 ± 3.93	37.34–42.33	0.209
12	40.42 ± 3.12	38.44–42.40	0.095
18	39.17 ± 3.51	36.94–41.40	0.341
24	39.92 ± 3.73	37.55–42.29	0.077
36	39.17 ± 3.61	36.87–41.46	0.198

CI: confidence interval; LLD: liver long diameter.

**Table 3:** Liver short diameter measurements at different postmortem time points.

Hour	Mean LSD (mm) ± SD	95% CI	*p* Value
0	15.67 ± 1.51	14.89–16.44	Reference
2	17.50 ± 1.62	16.59–18.41	0.005
4	17.75 ± 1.66	16.81–18.69	0.003
6	17.33 ± 1.56	16.45–18.21	0.005
9	16.92 ± 1.52	16.18–17.67	0.275
12	16.78 ± 1.34	16.12–17.43	0.102
18	16.44 ± 1.45	15.72–17.16	0.430
24	15.91 ± 1.37	15.27–16.55	0.012
36	15.33 ± 1.30	14.72–15.94	0.000

CI: confidence interval; LSD: liver short diameter.

**Table 4:** Spleen long diameter measurements at different postmortem time points.

Hour	Mean SLD (mm) ± SD	95% CI	*p* Value
0	17.08 ± 1.61	16.08–18.07	Reference
2	18.33 ± 1.72	17.23–19.42	0.011
4	18.58 ± 1.67	17.51–19.64	0.003
6	18.58 ± 1.24	17.79–19.37	0.002
9	18.41 ± 1.24	17.62–19.20	0.013
12	17.58 ± 1.16	16.84–18.32	0.236
18	17.33 ± 0.98	16.70–17.95	0.571
24	17.08 ± 1.08	16.39–17.77	1.000
36	16.91 ± 0.99	16.28–17.55	0.754

CI: confidence interval; SLD: spleen long diameter.

**Table 5:** Spleen short diameter measurements at different postmortem time points.

Hour	Mean SSD (mm) ± SD	95% CI	*p* Value
0	7.67 ± 0.62	7.17–8.16	Reference
2	7.91 ± 1.16	7.17–8.65	0.33
4	8.25 ± 0.96	7.63–8.86	0.002
6	8.50 ± 0.52	8.16–8.83	0.002
9	8.33 ± 0.65	7.91–8.74	0.005
12	7.66 ± 1.07	6.98–8.34	1.000
18	7.50 ± 0.67	7.07–7.92	0.586
24	7.41 ± 0.79	6.91–7.92	0.429
36	7.08 ± 0.66	6.65–7.50	0.089

CI: confidence interval; SSD: spleen short diameter.

**Table 6:** Liver SWE measurements at different postmortem time points.

Hour	Mean Liver SWE (kPa) ± SD	95% CI	*p* Value
0	32.34 ± 5.87	28.61–36.08	Reference
2	31.29 ± 8.50	25.89–36.70	0.57
4	31.96 ± 6.54	27.81–36.12	0.82
6	26.89 ± 6.83	22.55–31.24	0.003
9	26.50 ± 7.10	21.99–31.02	0.000
12	25.75 ± 5.84	22.04–29.47	0.004
18	23.87 ± 3.89	21.40–26.34	0.001
24	21.15 ± 3.89	18.67–23.62	<0.001
36	21.46 ± 5.75	17.80–25.12	0.001

CI: confidence interval; SWE: shear wave elastography; kPa: kilopascal.

**Table 7:** Spleen SWE measurements at different postmortem time points.

Hour	Mean Spleen SWE (kPa) ± SD	95% CI	*p* Value
0	33.09 ± 4.83	30.02–36.16	Reference
2	33.07 ± 4.51	30.20–35.94	0.994
4	31.88 ± 7.49	27.12–36.64	0.675
6	28.49 ± 7.27	23.87–33.12	0.064
9	24.56 ± 5.25	21.22–27.90	0.001
12	24.30 ± 4.40	21.51–27.10	0.001
18	24.27 ± 3.64	21.95–26.59	<0.001
24	22.50 ± 4.55	19.61–25.39	0.001
36	21.15 ± 4.58	18.24–24.06	<0.001

CI: confidence interval; SWE: shear wave elastography; kPa: kilopascal.

**Table 8:** Logistic regression analysis of shear wave elastography parameters for postmortem time estimation.

Parameter	β (Beta)	OR (Exp(B))	95% CI for OR (Lower–Upper)	*p* Value
Spleen SWE	−0.237	0.789	0.672–0.927	0.006
Liver SWE	−0.171	0.843	0.732–0.972	0.020

The binary logistic regression model was statistically significant (χ^2^ = 24.811, *p* < 0.001) with a classification accuracy of 75.0%, sensitivity of 79.2%, and specificity of 70.8% (Nagelkerke R^2^ = 0.538). SWE: shear wave elastography; β (Beta): regression coefficient; OR (Exp(B)): odds ratio; CI: confidence interval.

## Data Availability

The data that support the findings of this study are available from the corresponding author upon reasonable request.
